# Acupuncture for chronic persistent asthma based on the theory of Meridian-viscera Association: study protocol for a multi-center randomized controlled trial in China

**DOI:** 10.1186/s13063-020-04844-8

**Published:** 2021-01-06

**Authors:** Shirui Cheng, Xiaohui Dong, Lei Lan, Zhaoxuan He, Siyi Yu, Yi Yang, Chuantao Zhang, Mei Chen, Jun Yang, Haoran Chu, Yalan Liu, Menglin Wang, Qingsong Huang, Fang Zeng

**Affiliations:** 1 Acupuncture and Tuina School/The 3rd Teaching Hospital, Chengdu University of Traditional Chinese Medicine, 37# Shierqiao Road, Chengdu, 610075 Sichuan China; 2 Acupuncture & Brain Research Center, Chengdu University of Traditional Chinese Medicine, 37# Shierqiao Road, Chengdu, 610075 Sichuan China; 3 School of Administration, Chengdu University of Traditional Chinese Medicine, Chengdu, 610075 Sichuan China; 4 The First Affiliated Hospital of Chengdu University of Traditional Chinese Medicine, 39# Shierqiao Road, Chengdu, 610075 Sichuan China; 5 The Fifth People’s Hospital of Chengdu, Chengdu, 611130 Sichuan China; 6 The First Affiliated Hospital of Anhui University of Traditional Chinese Medicine, Hefei, Anhui China; 7 The Second Affiliated Hospital of Anhui University of Traditional Chinese Medicine, Hefei, 230031 Anhui China

**Keywords:** Acupuncture, Chronic persistent asthma, Randomized controlled trial, Protocol, Theory of Meridian-viscera Association

## Abstract

**Background:**

Acupuncture is effective in symptom and quality of life improvement of chronic asthma, but the efficacy differences between different acupoints are uncertain. In terms of the theory of Meridian-viscera Association, the study aims to investigate the different effectiveness between acupoints in Lung meridian and the acupoints in Heart meridian, so as to provide the evidence to develop a better prescription of the acupuncture treatment of chronic persistent asthma.

**Methods:**

This study is a multicentral randomized controlled trial. A total of 68 chronic persistent asthma patients will be randomly allocated into two groups: the Lung meridian group and the Heart meridian group. This trial will include a 2-week baseline period, a 4-week treatment period with 12 sessions’ acupuncture, and an 8-week follow-up period. The primary outcome is the Asthma Quality of Life Questionnaire (AQLQ). Secondary outcomes are the Asthma Control Test (ACT), Peak Expiratory Flow (PEF), and Forced Expiratory Volume in 1 s (FEV1). The AQLQ and ACT will be collected at baseline, week 4, week 8, and week 12 after randomization. PEF, FEV1, the Self-rating Anxiety Scale (SAS), and the Self-rating Depression Scale (SDS) will be assessed at baseline and week 4.

**Discussion:**

The results will provide evidence for acupuncture prescription selection and the clinical efficacy improvement. The results of this trial will also be used to determine whether or not a full definitive trial will go ahead, which will further confirm the theory of Meridian-viscera Association.

**Trial registration:**

Chinese Clinical Trial Registry (http://www.chictr.org.cn/showproj.aspx?proj=43803) ChiCTR1900027284. Registered on 7 November 2019

**Supplementary information:**

**Supplementary information** accompanies this paper at 10.1186/s13063-020-04844-8.

## Background

Asthma is characterized by bronchial hyper-responsiveness and chronic airway inflammation and accompanied by episodic wheezing, chest tightness, breathlessness, and cough [1, 2]. Asthma is one of the most common chronic respiratory diseases, and its prevalence varied from 0.2 to 21% in different countries [3]. The global prevalence of doctor-diagnosed asthma in adults is 4.3% and estimated to affect more than 300 million individuals worldwide [2]. Asthma not only significantly reduces patients’ quality of life (QoL) [4], leads to substantial disability [5], but also brings a heavy economic burden to patients, their families, and society, especially in low-income and middle-income countries [1, 6, 7]. Asthma is among the leading causes of mortality and morbidity worldwide [5], ranked among the top 20 conditions causing disability globally and ranked the 23rd as causes of disease burden as measured by disability-adjusted life years in 2015 [8, 9].

According to the 2019 Global Initiative for Asthma (GINA) guidelines on the management of asthma [2], current treatment options include asthma education, self-management strategies, elimination of risk factors, and medication control. Pharmacotherapies such as daily inhaled corticosteroids, long-acting inhaled β2-agonists are recommended to control symptoms and reduce the risk of serious exacerbations [2]. However, long-term pharmacological treatment inevitably has some potential side effects, such as adrenal suppression [10], decreased bone mineral density [11], liver toxicity [12], and increased risk of asthma-related death [13]. Therefore, seeking safe and effective therapies for asthma prevention and control aroused increasing concern [14–16].

Acupuncture, as a main complementary and alternative therapy, has traditionally been used to control asthma in China for thousands of years. Recently, accumulating evidence has supported the benefit of acupuncture for asthma [17, 18]. It is reported that acupuncture can significantly relieve the asthma-related symptoms, improve QoL, influence inflammatory cell counts [17], ameliorate of peak expiratory flow (PEF) variability [14], and increase the forced expiratory volume in 1 s (FEV1) [19]. However, most of these studies have focused on whether acupuncture is useful for asthma by comparing the efficacy differences between acupuncture and medication intervention [14, 18], or verum acupuncture and sham acupuncture [15, 17]. Few trials have involved the comparison of different acupuncture prescriptions to verify the efficacy differences between different acupuncture interventions.

According to the theory of traditional Chinese acupuncture, the Lung meridian connects with the lung directly, while the Heart meridian associates with the lung indirectly. Both the acupoints on Lung meridian and on the Heart meridian are commonly used for preventing and controlling asthma in clinical practice [20]. Hence, this multicenter randomized controlled trial focuses on the efficacy differences of the two acupoint prescriptions for treating asthma by evaluating the effectiveness of the acupoints on the Lung meridian compared to the acupoints on the Heart meridian, respectively, so as to provide high-quality evidence for a better acupuncture prescription selection and improve the curative effect.

## Method and design

### Study design

**Fig. 1:**
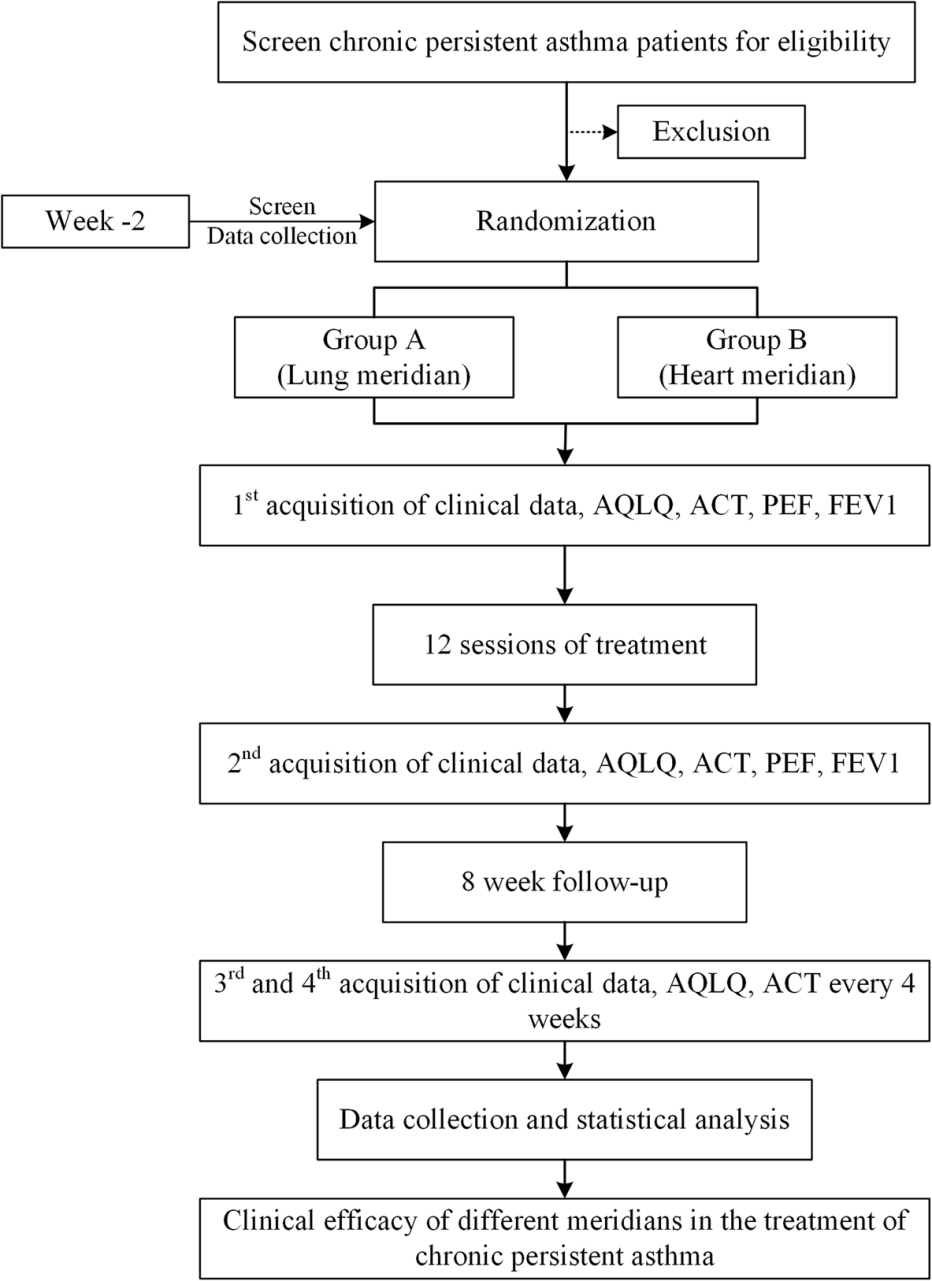
Study flowchart. Eligible patients will be randomly allocated into 2 groups with 1:1 ratio. Clinical data will be acquired at four timepoints: baseline (week 0), the end of treatment (week 4), and at the follow-up period (week 8, week 12). *AQLQ* Asthma Quality of Life Questionnaire, *ACT* Asthma Control Test, *PEF* Peak Expiratory Flow, *FEV1* Forced Expiratory Volume in 1 s

A two-arm, multi-center, randomized controlled trial will be conducted at the First Affiliated Hospital of Chengdu University of Traditional Chinese Medicine (CDUTCM) and the First Affiliated Hospital of Anhui University of Traditional Chinese Medicine. Sixty-eight eligible asthma patients diagnosed with the guide for asthma management and prevention of updated GINA 2019 [2] will be recruited and randomized into two groups with a 1:1 ratio. Outcome assessment will be performed at the baseline and the end of treatment and during the follow-up period. Patients enrolment will be started in November 2019 and is expected to end in October 2021. The details of the study design are shown in Fig. 1.

This trial is reported in accordance with the Standard Protocol Items: Recommendations for Intervention Trials (SPIRIT) guidelines [21] (Table 1, Additional file 1) and follows the principles of the Consolidated Standards of Reporting Trials (CONSORT) and Standards for Reporting Interventions in Clinical Trial of Acupuncture (STRICTA) [22]. The study has been approved by the Ethics Committee of the First Affiliated Hospital of CDUTCM (the approved number: 2019KL-045) and was registered at the Chinese Clinical Trial Registry (registration number: ChiCTR1900027284).

**Table 1:**
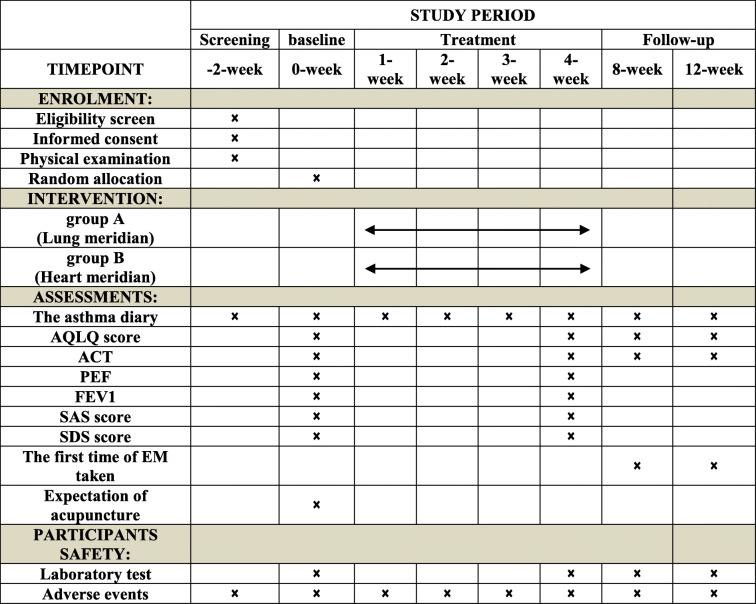
Standard Protocol Items: Recommendations for Interventional Trials (SPIRIT) schedule of the trial

This is a randomized controlled trial which includes a 2-week baseline period, a 4-week treatment period, and an 8-week follow-up period. In the baseline period, recruited patients will be screened, and eligible asthma patients will sign an informed consent and receive a physical examination. After allocation, the patients will receive 12 sessions of acupuncture (acupoint of Lung meridian or Heart meridian) during the treatment period. The outcome assessments, AQLQ, and ACT are performed at baseline, at the end of treatment, during the follow-up, and at the end of the follow-up. The outcome assessments, PEF, FEV1, SAS, and SDS are performed at baseline and at the end of treatment. In addition, the asthma dairy should be recorded at any time during the trial if asthma attacks. The first time of emergency medicine taken should be recorded at any time during the follow-up period. The expectation of acupuncture will be assessed at baseline. The physical examination including blood routine test and blood biochemical test will also be performed at the end of treatment to evaluate risks correlated with acupuncture. Adverse events will be recorded at any time during treatment

*AQLQ* Asthma Quality of Life Questionnaire, *ACT* Asthma Control Test, *PEF* Peak Expiratory Flow, *FEV1* Forced Expiratory Volume in 1 s, *SAS* Self-rating Anxiety Scale, *SDS* Self-rating Depression Scale, *EM* Emergency Medicine

### Randomization and allocation concealment

We will use an online or messaging system performed by a clinical information management system (Beijing Bioknow Information Science & Technology Co. Ltd., China) to complete the randomization. When participant-recruiting staffs in sub-center accept an eligible asthma patient, they will enter the patient’s name, gender, age, and medical history in the central randomization system. Then, a random number and an identification code, which are unique for each participant, will be assigned and delivered to the acupuncturists. The allocation number will be concealed from all the participants.

### Blinding

The allocation will be concealed from all the participants. Patients will be told that they will receive one of the two effective interventions randomized after enrolment. As patients in the two groups will adopt different acupoints, interventions of each patient will be performed in separated cubicles to refrain the communication among patients in the two groups and will be asked to wear an eye-patch when they receive treatment. Outcome assessors, data collectors, and statisticians will also be blind to the procedure and result of randomization, group allocation, and intervention. We do not anticipate any requirement for unblinding as the acupuncturist is not blinded, but if required, the Trial Manager, Data Coordinator will have access to group allocations and any unblinding will be reported.

### Participants and recruitment strategy

Participants will be mainly recruited from inpatients and outpatients in the respiratory department of the First Affiliated Hospital of CDUTCM, the Fifth People’s Hospital of Chengdu and the First Affiliated Hospital of Anhui University of Traditional Chinese Medicine. The recruitment strategies also include delivering leaflets at outpatients, posting advertisements in communities, and distributing news on the website of the Chengdu University of TCM and our WeChat public account. All potential asthma patients diagnosed with the GINA criteria will take a physical examination, which includes an x-ray plate, lung function test, electrocardiograph, routine blood test, liver function test, kidney function test, blood immune test (IgE), and urine and feces routine tests. The respiratory physician will make the final diagnosis of potential patients.

### Sample size

According to a previous study that investigates the therapeutic effect of acupuncture for asthma [23], the mean improvement of the AQLQ was − 2.75 ± 4.52 (mean ± SD) in the acupuncture group and 0.86 ± 4.95 in the control group. With a two-side significance level of α = 0.05 and power of 80%, 27 participants are required in each group. Allowing for a 20% dropout rate, 34 participants per treatment arm will be recruited, equaling 68 patients in total.

### Inclusion criteria

Patients fulfilling the following 7 items will be included: (1) aging from 18 to 65 years, (2) diagnosed as chronic persistent asthma by respiratory physicians according to the guide of GINA 2019 and medical history, (3) having mild or moderate asthma according to the GINA 2019, (4) having increased in the FEV1 of > 12% and > 200 mL from the pre-bronchodilator value, (5) the FEV1/forced vital capacity (FVC) < 70%, (6) without participating in any other clinical trials or receiving acupuncture due to asthma within 1 month, and (7) having signed informed consent.

### Exclusion criteria

Patients who match any of the following 8 items will be excluded: (1) be hospitalized in intensive care unit due to acute exacerbation of asthma in the previous 3 months (representing patients at highest risk of adverse asthma outcomes); (2) be diagnosed as other pulmonary diseases such as bronchiectasis, tuberculosis, abscess, cystic fibrosis, alpha 1 antitrypsin deficiency, or restrictive lung disease; (3) having serious cardiovascular diseases such as uncontrolled heart failure, coronary disease, myocardial infarction, severe hypertension, or uncontrolled arrhythmia; (4) having other uncontrolled diseases including liver or kidney or hematopoietic diseases, endocrine and immune diseases, mental disorders, and malignancy; (5) asthma occurs only when accidentally exposed to allergens or chemical sensitizers; (6) having lower respiratory tract infection (e.g., pneumonia) or taking systemic glucocorticoids more than 10 days for severe acute asthma attacks within 1 month before enrollment; (7) currently be pregnant or breast feeding at the time of enrolment or planned pregnancy within the study period; or (8) having any acupuncture contraindications, such as serious atopic, infectious dermatopathy, thrombocytopenic purpura, or hemophilia.

### Acupuncture interventions

The acupuncture treatment of each group will consist of 12 sessions of 30 min duration, each administered over a period of 4 weeks (three sessions per week). All the acupuncture manipulation will be performed by licensed acupuncturists with at least 5 year’s clinical experience.

**Fig. 2:**
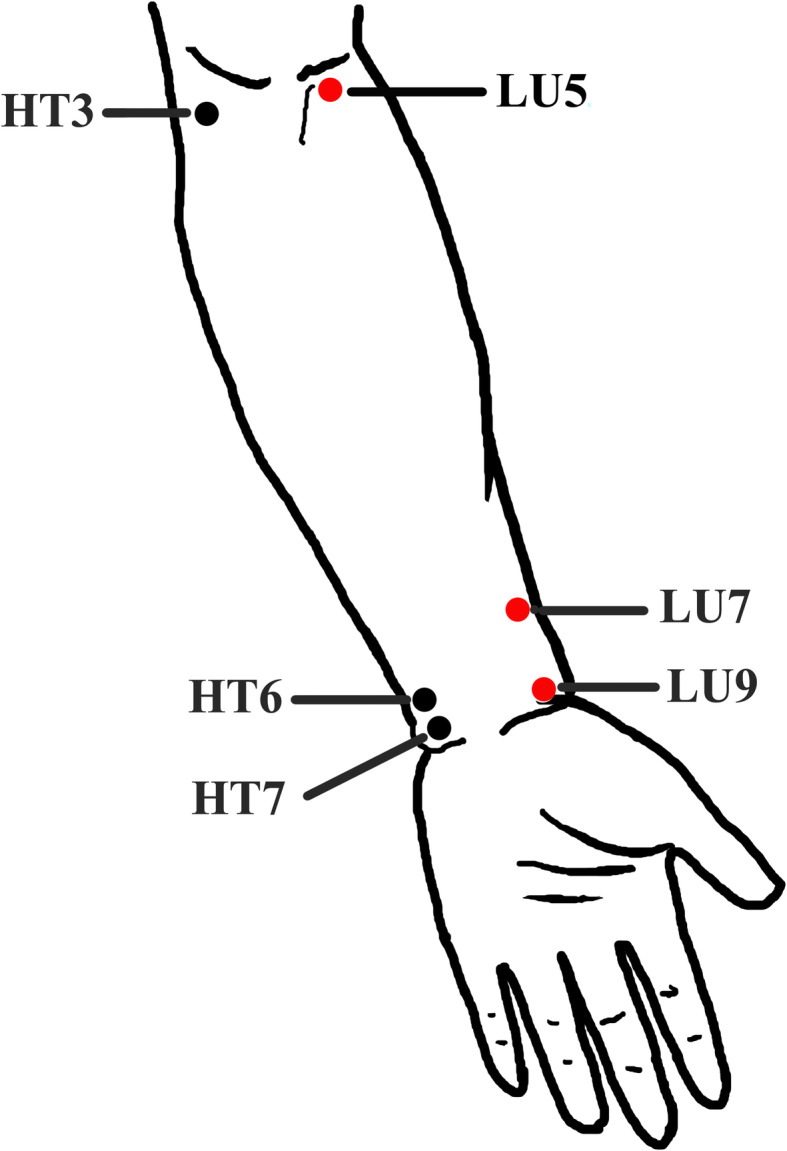
Locations of acupoints. *Acupoints on Lung meridian—*Chize (LU5): on the cubital crease, on the radial side of the tendon of biceps brachii; Lieque (LU7): on the radial margin of the forearm, 1.5 cun above the transverse crease of the wrist, between the brachioradial muscle tendon and the long abductor muscle tendon of thumb; and Taiyuan (LU9): on the radial side of the transverse crease of the wrist, where the radial artery pulsates. *Acupoints on Heart meridian*—Shaohai (HT3): at the midpoint of the line connecting the medial end of the transverse cubital crease with the medial epicondylus of the humerus; Yinxi (HT6): on the palmer side of the forearm, the point is on the radial side of the tendon of the flexor carpi ulnaris, 0.5 cun above the transverse crease of the wrist; and Shenmen (HT7): on the wrist, at the ulnar end of the transverse crease of the wrist, in the depression on the radial side of the tendon of the flexor carpi ulnaris

The manual acupuncture stimulation will be performed at bilateral Taiyuan (LU9), Lieque (LU7), and Chize (LU5) for patients in group A, and manual acupuncture stimulation will be performed at bilateral Shenmen (HT7), Yinxi (HT6), and Shaohai (HT3) for patients in group B (Fig. 2).

The acupuncture manipulation of these two groups is as follows: the disposable sterile filiform needles (0.25 × 25 mm, 0.35 × 40 mm; Huatuo Medical Instrument Co., Ltd., China) will be inserted into acupoints with the depth of 10–30 mm after skin disinfection by alcohol. Manipulations of twirling, lifting, and thrusting will be performed in order to induce *deqi* sensation. Then needles will be retained at the acupoints for 30 min. In order to maintain the *deqi* sensation, the above procedures will be manipulated intermittently during the 30 min.

### Medications

Patients can use the inhaled doctor-directed Seretide (salmeterol and fluticasone) or Symbicort (formoterol and budesonide) as conventional therapy following the respiratory physicians’ instruction. Participants will be not allowed using other interventions during this trial but will be permitted to use inhaled salbutamol or other medication advised by doctors in an emergency. The name of medications, specifications, dose, duration of use, time of symptom relief, etc. will be recorded in details.

There should be at least 6 h between the use of salbutamol and lung function measurements. A stable dose of inhaled glucocorticoids and antihistamines should be maintained for at least 2 months prior to enrollment and during this study. If patients’ desensitization therapy is in the “maintenance” phase, the medication used should be maintained at a stable dose of at least 1 month prior to the enrollment and during this study. Short courses of antihistamine therapy or inhaled glucocorticoid therapy are allowed to treat hay fever. Short-term (< 10 days) use of systemic corticosteroids, aerosol therapy (including β2-agonists, anticholinergics, and steroids), and antibiotic therapy will be permitted during this study.

### Outcome measurements

#### Primary outcome

##### Asthma Quality of Life Questionnaire (AQLQ)

In this study, the standard version of AQLQ will be used [24]. This questionnaire measures the functional problems that are most troublesome to adults with asthma over the 2 weeks prior to the interview. The AQLQ contains 32 items: activity limitation (11 items), symptoms (12 items), mental health (5 items), and environmental stimuli (4 items). Responses are rated on 7-point Likert scales for every item. High scores indicate a better health-related quality of life. The mean score of all 32 items is the overall AQLQ score. The minimum clinically important difference for AQLQ has been reported as 0.52 [25].

#### Secondary outcomes

##### Asthma Control Test (ACT)

Asthma control will be evaluated using the Asthma Control Test (ACT) [26, 27]. The ACT consists of five items: activity limitation, daytime shortness of breath, awaking due to asthma symptoms, needed puffs of reliever medication, and a global judgment of asthma control. All items refer to the last 4 weeks. They are scaled from 1 to 5. The sum of scores indicates asthma control. An ACT score of 20–25 indicates controlled asthma and of < 20 indicates uncontrolled asthma. A minimum clinically important difference of about 3 was identified [28].

##### Peak Expiratory Flow (PEF) and Forced Expiratory Volume in 1 s (FEV1)

PEF and FEV1 are determined using spirometry and body plethysmography before and after bronchodilation with a short-acting bronchodilator in accordance with recommendations of the national guidelines [29, 30].

The Self-rating Anxiety Scale (SAS) [31] and the Self-rating Depression Scale (SDS) [32] are used to assess the emotion of asthma patients. In addition, the asthma diary including the times, severity, duration of asthma attacks, medication compliance during the trial, and the first time to use the emergency medicine after treatment will also be recorded.

AQLQ and ACT will be evaluated at baseline (week 0), after the intervention (week 4), and at the follow-up (week 8, week 12). The PEF, FEV1, SAS, and SDS will be assessed at baseline (week 0), after the intervention (week 4). The asthma diary will be assessed during the whole trial (week 0– week 12). The expectation for efficacy in patients will be evaluated at baseline [33]. The first time to use emergency medicine after treatment will be evaluated during the follow-up. All the outcome evaluations will be performed by two independent outcome assessors. The two assessors are trained before participating in this trial and blinded to the randomization.

### Data monitoring and management

All data will be managed with printed and electronic Case Report Forms (CRFs). Primary entries are not allowed to be changed and any correction should be explained with a signature in the appended notes. Only outcome assessors have access to CRFs, and double-data entry will be performed. The research team in Chengdu is responsible for all aspects of the organization. The Ethics Committee of the First Affiliated Hospital of Chengdu University of TCM will be supervising this trial and data every 3 months and will make the final decision to terminate the trial. The process will be independent from investigators and the sponsor. Interventionists will monitor adverse events and respond appropriately with encouragement and suggestions. All researchers, physicians, and related personal are required to understand and adhere to protocol details.

### Data analysis

All data will be analyzed by an independent statistician using SAS 9.4 software (SAS Institute Inc., Cary, NC). All efficacy and safety analyses will be strictly conducted according to the intention-to-treat (ITT) principle. Multiple imputation will be used to deal with missing data. Continuous variables will be described with means and standard deviation (SD) or median, maximum, minimum, P25, and P75, as appropriate. And categorical variables will be described with percentage and frequencies.

The comparison between groups will be analyzed by the analysis of covariance (ANCOVA) if the change of outcomes from baseline to 4 weeks is normally distributed. The outcome variable will be the change of AQLQ from baseline to week 4, and covariates will be age, gender, duration of disease, and the expectation of acupuncture in the ANCOVA analysis. The non-parametric ANCOVA will be conducted if the change is not normally distributed. The comparisons between time points within groups will be analyzed using the paired sample *t* test or Wilcoxon rank-sum test, as appropriate. Separate ANCOVA analyses will be performed for each secondary outcome, the AQLQ and ACT at week 8 and week 12 follow-up. Categorical variables will be analyzed using the chi-squared test or Fisher’s exact test, as appropriate.

The center effects will be included in the analysis model. A subgroup analysis between inpatient and outpatient participants in each group will be performed exploratively. Statistical results will be presented as point estimates of effect sizes accompanied by confidence intervals.

### Patient safety

Participants with fainting, infection, or other severe adverse events should be discontinued from treatment and processed immediately. Any adverse events will be recorded by patients, acupuncturists, and outcome assessors. Acupuncture related adverse events include bleeding, dizziness, pain, infection, and so on. Besides, any other severe events also will be recorded in the CRFs in detail and be monitored by the Ethics Committees.

### Quality control

The trial protocol is reviewed and revised by the experts in acupuncture, methodology, pneumology, and statistics. The related staff will be trained using a prespecified standard operating procedure. The compliance of patients will be improved through health education and timely follow-up. The outcomes will also be evaluated by outcome assessors if the participants discontinue acupuncture or deviate from protocol.

## Discussion

This is the first RCT focused on the efficacy differences of the acupoints on the Lung meridian compared to the acupoints on the Heart meridian for treating chronic persistent asthma. The results will provide evidence for acupuncture prescription selection and the clinical efficacy improvement. The results of this trial will also be used to determine whether or not a full definitive trial will go ahead, which will further confirm the theory of Meridian-viscera Association.

The theory of Meridian-viscera Association emphasizes the diagnostic and therapeutic values based on the mutual relation in physiology and pathology between meridians and viscera. That is, visceral physiological functions and pathological changes will manifest in the corresponding meridians or the acupoints [34], and the visceral disorders can be treated with the involved meridian and acupoints [35]. The lung is close to the heart anatomically, so the two viscera have close association physio-pathologically. Based on the theory of Meridian-viscera Association and clinical practice, the Lung meridian and the Heart meridian are the commonly used meridians for pulmonary disorders including asthma [17, 18]. Hence, this study will select Taiyuan (LU9), Lieque (LU7), and Chize (LU5) on the Lung meridian and Shenmen (HT7), Yinxi (HT6), and Shaohai (HT3) on the Heart meridian for the following reasons: (1) these acupoints have been proved to be effective in improving QoL and relieving symptoms of asthma patients [18, 23], (2) all of these acupoints are specific acupoints on the two meridians, and (3) the selected acupoints are on the medial side of the upper arm and at the same nerve segment based on the modern anatomy so as to minimized the bias of comparison.

The efficacy evaluation will be performed from the following three aspects: the quality of life, symptoms, and emotion of asthma patients. Asthma affects patients’ daily life seriously [4]; the improvement of QoL is essential for asthma patients, especially chronic persistent asthma patients. So the AQLQ will be assessed as the primary outcome, which is a reliable, valid, discriminating, and responsive measurement and is more sensitive than the 36-Item Short Form Survey (SF-36) questionnaire in detecting differences in asthma severity [36]. At the same time, the ACT, PEF, and FEV1, as secondary outcomes, will be evaluated before and after treatment in this trial. Patients with poor-controlled or uncontrolled asthma have more serious symptoms and worse lung function [37]. Hence, ACT will be performed to identify patients with poorly controlled asthma [26, 27], and PEF and FEV1 will be used to assess the severity of lung function in asthma patients [29, 30]. Moreover, anxiety and depression are emotional common disorders accompanied by adult asthma [38, 39]. So the SAS [31] and SDS [32] are used to assess the emotion of asthma patients.

### Trial status

This trial is registered on the Chinese Clinical Trial Registry (http://www.chictr.org.cn) on 7 November 2019 (Registered number: ChiCTR1900027284, the protocol version number: V2.0). The patient recruitment of this trial has not been started and will begin on November 25, 2019, and will be completed on October 31, 2021.

## Supplementary Information

**Additional file 1.** SPIRIT 2013 Checklist: Recommended items to address in a clinical trial protocol and related documents.

## Data Availability

The data and materials during the current study are available from the corresponding author on reasonable request.

## Abbreviations

ACT: Asthma Control Test
ANCOVA: The analysis of covariance
AQLQ: Asthma Quality of Life Questionnaire
CDUTCM: Chengdu University of Traditional Chinese Medicine
CONSORT: The Consolidated Standards of Reporting Trials
CRFs: Case Report Forms
FEV1: Forced Expiratory Volume in 1 s
FVC: Forced Vital Capacity
GINA: Global Initiative for Asthma
ITT: Intention-to-treat analysis
PEF: Peak Expiratory Flow
QoL: Quality of Life
RCT: Randomized Controlled Trial
SAS: Self-rating Anxiety Scale
SD: Standard Deviation
SDS: Self-rating Depression Scale
SF-36: 36-Item Short Form Survey
SPIRIT: Standard Protocol Items Recommendations for Intervention Trials
STRICTA: Standards for Reporting Interventions in Clinical Trial of Acupuncture
TCM: Traditional Chinese Medicine
